# Severe allergic reaction to diethyltoluamide (DEET) containing insect repellent

**DOI:** 10.1186/1710-1492-10-S2-A30

**Published:** 2014-12-18

**Authors:** Mary McHenry, Gina Lacuesta

**Affiliations:** 1 Department of Pediatrics, Dalhousie University, Halifax, Nova Scotia, Canada; 2 Division of Allergy and Clinical Immunology, Dalhousie University, Halifax, Nova Scotia, Canada

## Background

Contact and generalized urticaria to DEET-containing repellents have been reported, but few cases of severe allergic reaction with angioedema.

## Case presentation

A 53 year-old female bridge inspector presented with allergic reaction to diethyltoluamide (DEET) - containing insect repellent. She had prior use without difficulty. In 2013, she used the insect repellent and with only a small amount making contact with her forehead, she had immediate pruritus and erythema on her forehead, persisting for an hour. The following week, she used a different insect repellent and sprayed her face and body. Within minutes, she became diffusely pruritic with generalized urticaria and angioedema of her eyes. She called 911 and was given intramuscular diphenhydramine. Her symptoms gradually eased and she was subsequently well. Her regular medications include venlafaxine and ketorolac. She has no history of atopy. Since the reaction, she has been avoiding all forms of insect repellent, including riding in separate vehicles as her co-workers who use insect repellent. She carries an epinephrine device at all times. Skin testing was performed using two DEET-containing insect repellents: Lloyd’s Bug Spray^©^ (23.75% DEET) and OFF Family Care Bug Spray^©^ (5% DEET). She had positive skin prick test to both insect repellents, more prominent with the higher containing DEET repellent. There was also a significant reaction on the skin adjacent to the test site where the repellent had not made contact. She developed significant pruritus and was treated with oral anti-histamine. She had appropriate controls. A control subject tested in the office was negative to both insect repellents.

## Conclusion

The patient had a severe allergic reaction to insect repellent, and exhibits sensitization based on skin testing. This represents a unique case of severe cutaneous reaction to insect repellent and such patients may be at risk of anaphylaxis with future exposure.

## Consent

Written informed consent was obtained from the patient for publication of this abstract and any accompanying images. A copy of the written consent is available for review by the Editor of this journal.

